# Musicians Are Better than Non-musicians in Frequency Change Detection: Behavioral and Electrophysiological Evidence

**DOI:** 10.3389/fnins.2016.00464

**Published:** 2016-10-25

**Authors:** Chun Liang, Brian Earl, Ivy Thompson, Kayla Whitaker, Steven Cahn, Jing Xiang, Qian-Jie Fu, Fawen Zhang

**Affiliations:** ^1^Department of Communication Sciences and Disorders, University of CincinnatiCincinnati, OH, USA; ^2^Department of Composition, Musicology, and Theory, College-Conservatory of Music, University of CincinnatiCincinnati, OH, USA; ^3^Department of Pediatrics and Neurology, Cincinnati Children's Hospital Medical CenterCincinnati, OH, USA; ^4^Department of Head and Neck Surgery, University of California, Los AngelesLos Angeles, CA, USA

**Keywords:** frequency change detection, auditory evoked potentials, acoustic change complex, electrophysiology, cortex

## Abstract

**Objective:** The objectives of this study were: (1) to determine if musicians have a better ability to detect frequency changes under quiet and noisy conditions; (2) to use the acoustic change complex (ACC), a type of electroencephalographic (EEG) response, to understand the neural substrates of musician vs. non-musician difference in frequency change detection abilities.

**Methods:** Twenty-four young normal hearing listeners (12 musicians and 12 non-musicians) participated. All participants underwent psychoacoustic frequency detection tests with three types of stimuli: tones (base frequency at 160 Hz) containing frequency changes (Stim 1), tones containing frequency changes masked by low-level noise (Stim 2), and tones containing frequency changes masked by high-level noise (Stim 3). The EEG data were recorded using tones (base frequency at 160 and 1200 Hz, respectively) containing different magnitudes of frequency changes (0, 5, and 50% changes, respectively). The late-latency evoked potential evoked by the onset of the tones (onset LAEP or N1-P2 complex) and that evoked by the frequency change contained in the tone (the acoustic change complex or ACC or N1′-P2′ complex) were analyzed.

**Results:** Musicians significantly outperformed non-musicians in all stimulus conditions. The ACC and onset LAEP showed similarities and differences. Increasing the magnitude of frequency change resulted in increased ACC amplitudes. ACC measures were found to be significantly different between musicians (larger P2′ amplitude) and non-musicians for the base frequency of 160 Hz but not 1200 Hz. Although the peak amplitude in the onset LAEP appeared to be larger and latency shorter in musicians than in non-musicians, the difference did not reach statistical significance. The amplitude of the onset LAEP is significantly correlated with that of the ACC for the base frequency of 160 Hz.

**Conclusion:** The present study demonstrated that musicians do perform better than non-musicians in detecting frequency changes in quiet and noisy conditions. The ACC and onset LAEP may involve different but overlapping neural mechanisms.

**Significance:** This is the first study using the ACC to examine music-training effects. The ACC measures provide an objective tool for documenting musical training effects on frequency detection.

## Introduction

Frequency information is important for speech and music perception. The fundamental frequency (F0) is the lowest frequency of a periodic sound waveform. The F0 plays a critical role in conveying linguistic and non-linguistic information that is important for perceiving music and tone languages, differentiating vocal emotions, identifying a talker's gender, and extracting speech signals from background noise or competing talkers. Unfortunately, for hearing impaired listeners such as cochlear implant (CI) users, these frequency-based tasks are tremendously challenging due to the limitations of current CI technology (Kong and Zeng, 2004; Fu and Nogaki, 2005; Stickney et al., 2007; Zeng et al., 2014).

Considerable evidence has shown that hearing-impaired listeners achieve maximal benefit from brain plasticity as a result of auditory training, such that the auditory system can be more sensitive to the poorer neural representation of acoustic information at the peripheral auditory system (Fu et al., 2004; Galvin et al., 2009; Lo et al., 2015). The potential benefit of auditory training with music stimuli has drawn increasing attention from researchers in recent years (Looi et al., 2012; Gfeller et al., 2015; Hutter et al., 2015) because of the following reasons: (1) music training, such as the training experienced by musicians, may positively enhance speech perception due to the physical features (e.g., frequency, rhythm, intensity, and duration) and overlapping neural networks for processing between the two stimuli (Patel, 2003; Besson et al., 2007; Kraus et al., 2009; Parbery-Clark et al., 2009; Petersen et al., 2009; Itoh et al., 2012), (2) music training enhances cognitive functions, which are required for both language and music perception (Strait et al., 2010, 2012; Strait and Kraus, 2011; Kraus, 2012), and (3) some behavioral data showed that the performance in hearing impaired listeners significantly improves with music training (Gfeller et al., 2015; Hutter et al., 2015). These findings suggest that music training may be integrated into cross-cultural nonlinguistic training regimens to alleviate perceptual deficits in frequency-based tasks for hearing impaired patients. Therefore, further understanding of the effects of music training has significant implications in pointing to the direction of auditory rehabilitation for hearing impaired listeners.

Numerous studies have examined music training effect through musician vs. non-musician comparisons, because musicians' brains serve as excellent models to show brain plasticity as a result of routine music training. In the auditory domain, musicians have superior auditory perceptual skills and their brains can better encode frequency information (Koelsch et al., 1999; Parbery-Clark et al., 2009). Most sounds in our environment including speech and music contain frequency changes or transitions, which are important cues for identifying and differentiating these sounds. Most previous studies examining pitch perception in musicians vs. non-musicians used a frequency discrimination task that focuses on the detection of one frequency that is different from the reference frequency (Tervaniemi et al., 2005; Micheyl et al., 2006; Bidelman et al., 2013) rather than the detection of the frequency change contained in an ongoing stimulus. In such a discrimination task, the auditory system needs to detect the individual sounds at different frequencies, thereby the neural mechanism may involve the detection of the onset of the sounds at different frequencies rather than the frequency change *per–se*. Therefore, the frequency discrimination task may not be optimal for the understanding of how the auditory system responds to frequency changes in a context. A frequency change detection task using the stimuli containing frequency changes may provide better insights about the underlying mechanisms of the auditory system responding to frequency changes in a context.

Auditory evoked potentials recorded using EEG techniques have been used to understand the neural substrates of frequency change detection. The late auditory evoked potential (LAEP) is an event-related potential reflecting central processing of the sound. The N1 peak of the LAEP occurs at a latency of ~100 ms and the P2 peak at a latency of 200 ms. The acoustic change complex (ACC) is a type of LAEP evoked by the acoustic change in an ongoing stimulus (Ostroff et al., 1998; Small and Werker, 2012). The ACC can be evoked by the consonant-vowel transition in an ongoing syllable (Ostroff et al., 1998; Friesen and Tremblay, 2006), the change of acoustic feature (e.g., frequency or amplitude, Martin and Boothroyd, 2000; Harris et al., 2007; Dimitrijevic et al., 2008), and the change in place of stimulation within the cochlea (i.e., in CI users, Brown et al., 2008). The minimal acoustic change that can evoke the ACC is similar to the threshold for auditory discrimination threshold (Harris et al., 2007; He et al., 2012). The ACC recording does not require participants' active participation and it provides an objective measure of stimulus differentiation capacity that can be used in difficult-to-measure subjects.

The ACC has been recorded reliably in normal hearing (NH) adults, young infants, hearing aid users, and CI users (Friesen and Tremblay, 2006; Tremblay et al., 2006; Martin, 2007; Kim et al., 2009; Small and Werker, 2012). However, the exact nature of the ACC has not been well understood. One unanswered question is: what are the differences between the ACC evoked by acoustic changes and the conventional LAEP evoked by stimulus onset? The previous studies used stimuli to evoke the ACC containing both onset of new stimulus compared to the base stimulus and acoustic changes (Itoh et al., 2012; Small and Werker, 2012) or stimuli containing acoustic changes in more than one dimension, e.g., the change in stimulation electrode in CI users and the change in perceived frequency due to the change in stimulation electrode (Brown et al., 2008) and the changes in spectral envelope, amplitude, and periodicity at the transition in consonant-vowel syllables (Ostroff et al., 1998; Friesen and Tremblay, 2006; Tremblay et al., 2006).

The current study will examine the musician benefit using frequency changes in the simplest tone, with the onset cues removed, for both behavioral tests of frequency change detection and EEG recordings. Through the combination of behavioral and EEG measures, the neural substrates underlying musician benefit in frequency change detection would be better understood. This information is critical for the design of efficient training strategies. The practical outcome of such research study would be that, if the music training effects can be reflected in EEG measures, the EEG measurement can be used for objective evaluation of music training effects. The objectives of this study were: (1) to determine if musicians have better ability to detect frequency changes in quiet and noisy conditions; (2) to use the ACC measure to understand the neural substrates of musicians vs. non-musicians in frequency change detection. To our knowledge, this is the first study using the ACC to examine music training effects in musicians.

## Materials and methods

### Subjects

Twenty-four healthy young NH individuals (13 males and 11 females; age range: 20–30 years) including 12 musicians and 12 non-musicians participated in the study. All participants had audiometric hearing thresholds ≤ 20 dB HL at octave test frequencies from 250 to 8000 Hz, normal type A tympanometry, and normal acoustic reflex thresholds at 0.5, 1, and 2 kHz. All participants were right-handed and did not have neurological or hearing-related disorders. The criteria for musicians were: (a) having at least 10 years of continuous training in Western classical music on their principal instruments, (b) having begun music training at or before the age of 7, (c) having received music training within the last 3 years on a regular basis. All of the 12 musicians were students from the College of Conservatory of Music at the University of Cincinnati; the instruments played by these musicians include piano, guitar saxophone, cello, trumpet, horn, and double bass. The criteria for non-musicians were: (a) having no more than 3 years of formal music training on any combination of instruments throughout their lifetime, (b) having no formal music training within the past 5 years. The above criteria for musicians and non-musicians are similar to the criteria used in previous studies (Bidelman et al., 2013; Fuller et al., 2014). All of the 12 non-musicians were college students with non-music majors. All participants gave informed written consent prior to their participation. This research was approved by the Institutional Review Board of the University of Cincinnati.

### Stimuli

#### Stimuli for behavioral tests

The stimuli were tones generated using Audacity 1.2.5 (http://audacity.sourceforge.net) at a sample rate of 44.1 kHz. A tone of 1 s duration at a base frequency of 160 Hz, which is in the frequency range of the F0 of the human voice, was used as the standard tone. To avoid an abrupt onset and offset, the amplitude was reduced to zero over 10 ms using the fade in and fade out function. The target tones were the same as the standard tone except that the target tones contained upward frequency changes at 500 ms after the tone onset, with the magnitude of frequency change varying from 0.05 to 65% (the large range was created so that the same stimuli could be used for CI users in a future study). The frequency change occurred for an integer number of cycles of the base frequency and the change occurred at 0 phase (zero crossing). If the number of base frequency cycles was not an integer at 500 ms, the number of cycles was rounded up to an integer number which leads to a slightly delayed point (not exactly at 500 ms) for the start of the frequency change. Therefore, the onset cue was removed and it did not produce audible transients (Dimitrijevic et al., 2008).

The above stimuli were mixed with broad band noise of the same duration to create two more sets of stimuli: tones containing frequency changes masked by low-level noise, and tones containing frequency changes masked by high-level noise. The onset- and offset-amplitude of the broad band noise was reduced to zero over 10 ms, the same as how the tones were treated. For the low-level noise, the root mean square (RMS) amplitude of the noise was 10 dB lower than that of the tone (SNR = 10 dB); for the high-level noise, the RMS amplitude of the noise was the same as that of the tone stimulus (SNR = 0 dB); The amplitudes of all stimuli were normalized. The stimuli were calibrated using a Brüel and Kjær (Investigator 2260) sound level meter set on linear frequency and slow time weighting with a 2 cc coupler.

For convenience, the stimuli for frequency detection tasks were renamed numerically: tone stimuli containing frequency changes (Stim 1), tones containing frequency changes masked by low-level noise (Stim 2), and tones containing frequency changes masked by high-level noise (Stim 3).

#### Stimuli for EEG recording

Tones of 160 and 1200 Hz with 1 s duration that contained upward frequency changes were used as stimuli for EEG recordings. These two different base frequencies were used for EEG recording for the following reasons. First, while 160 Hz is in the frequency range of the F0 of the human voice, 1200 Hz is in the frequency range of the 2nd formant of vowels. Examining the ACC at these two base frequencies would help understand how the auditory system processes the frequency change near the F0 and the 2nd formant of vowels; second, this would help us better understand the differences of the ACC and the onset LAEP. Specifically, the ACC is evoked by frequency change from the base frequency. But is the ACC evoked by a frequency change (e.g., a small change from 160 to 168 Hz for a 5% change) predictable using the onset LAEP evoked by the onset of different frequencies (160 vs. 1200 Hz)? The amount of the frequency change was manipulated at 0% (no change), 5, and 50%, respectively. Note, that the stimuli used for EEG recordings were presented in quite conditions. Therefore, the six stimuli (3 types of frequency changes × 2 base frequencies) were presented with 200 trials for each, with a randomized order. The inter-stimulus interval was 800 ms.

### Procedure

#### Behavioral tests of frequency change detection

The participants were comfortably seated in a sound-treated booth. Stimuli were delivered in the sound field via a single loudspeaker placed at ear level, 50 cm in front of the participant at the most comfortable level (7 on a 0–10 loudness scale). Such a presentation approach, which has been commonly used in CI users, was used so that the current data can be compared with those from CI users in a future study. The stimuli were presented using APEX (Francart et al., 2008). An adaptive, 2-alternative forced-choice procedure with an up-down stepping rule was employed to measure the minimum frequency change the participant was able to detect. In each trial, a target stimulus and a standard stimulus were included. The standard stimulus was the tone without frequency change and the target stimulus was the tone with a frequency change. The order of standard and target stimulus was randomized and the interval between the stimuli in a trial was 0.5 s. The participant was instructed to choose the target signal by pressing the button on the computer screen and was given a visual feedback regarding the correct response. Each run generated a total of five reversals. The asymptotic amount of frequency change (the average of the last three trials) then became an estimate of the threshold for frequency change detection. Each participant was required to do the frequency change detection task with the three types of stimuli (Stim 1, 2, and 3). The order of the three stimulus type conditions was randomized and counterbalanced across participants.

#### EEG recording

Participants were fitted with a 40-channel Neuroscan quick-cap (NuAmps, Compumedics Neuroscan, Inc., Charlotte, NC). The cap was placed according to the International 10–20 system, with the linked ear as the reference. Electro-ocular activity (EOG) was monitored so that eye movement artifacts could be identified and rejected during the offline analysis. Electrode impedances for the remaining electrodes were kept at or below 5 kΩ. EEG recordings were collected using the SCAN software (version 4.3, Compumedics Neuroscan, Inc., Charlotte, NC) with a band-pass filter setting from 0.1 to 100 Hz and an analog-to-digital converter (ADC) sampling rate of 1000 Hz. During testing, participants were instructed to avoid excessive eye and body movements. Participants read self-selected magazines to keep alert and were asked to ignore the acoustic stimuli. Participants were periodically given short breaks in order to shift body position and to maximize alertness during the experiment.

### Data processing

For the behavioral test, the frequency change detection threshold was measured for each of the three stimuli in each participant. For EEG results, continuous EEG data collected from each participant were digitally filtered using a band-pass filter (0.1–30 Hz). Then the data were segmented into epochs over a window of 1500 ms (including a 100 ms pre-stimulus duration). Following segmentation, baseline was corrected by the mean amplitude of the 100 ms pre-stimulus time window and epochs in which voltages exceeded ±150 μV were rejected from further analysis. Then EEG data were averaged separately for each of the six types of stimuli (2 base frequencies × 3 types of frequency changes) in each participant. Then, MATLAB (Mathworks, Natick, MA) was used to objectively identify peak components, which were confirmed by visual evaluation of the experimenters. Because the LAEP was largest at electrode Cz, we restricted the later analysis to data from Cz.

The onset LAEP response peaks were labeled using standard nomenclature of N1 and P2. The ACC response peaks were labeled using N1′ and P2′. The N1 and P2 peaks of the onset LAEP were identified in a latency range 70–180 and 150–250 ms, respectively, after the onset of the tone; The N1′ and P2′ peaks of the ACC were identified in a latency range 70–180 and 150–250 ms, respectively, after the onset of the frequency change. The measures used for statistical analysis include: N1 and P2 amplitude and latency, N1-P2 peak-to-peak amplitude for the onset LAEP and the corresponding measures for the ACC.

The series of mixed-design repeated analysis of variance (ANOVA) were performed to examine the difference in behavioral and EEG measures between the musician and non-musician groups under different stimulus conditions. Pearson correlation analysis was performed to determine if ACC measures correlate to onset LAEP measures, and if behavioral frequency detection thresholds correlate to ACC measures. A *p*-value of 0.05 was used as the significance level for all analyses.

## Results

### Psychoacoustic performance

**Figure 1:**
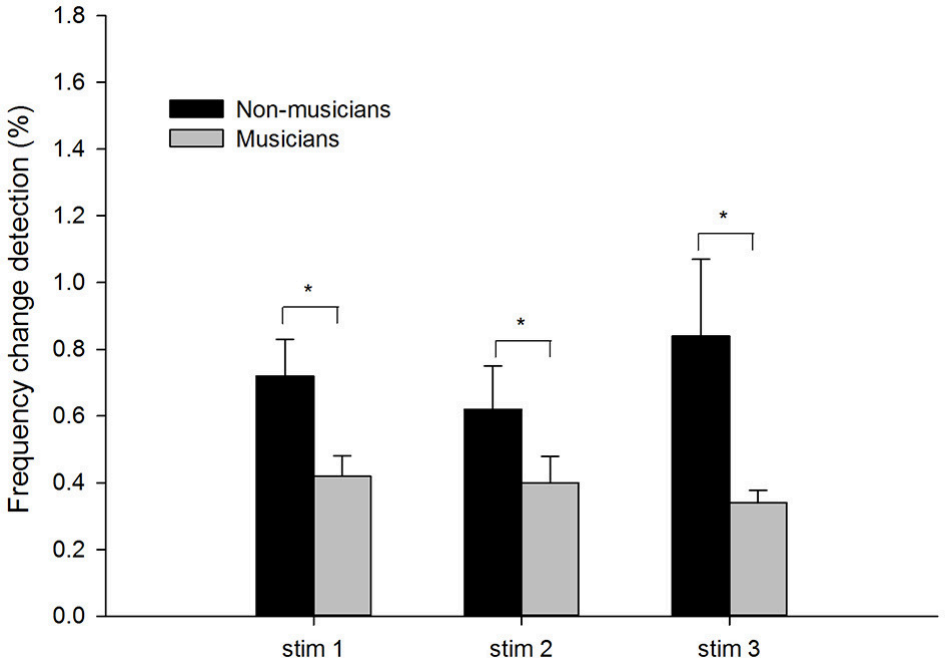
**The frequency detection thresholds of musicians and non-musicians for the three stimulus conditions: tone stimuli containing frequency changes (Stim 1), tones containing frequency changes with low-level noise (Stim 2), and tones containing frequency changes with high-level noise (Stim 3)**. The error bars indicate standard errors of the means. Asterisks denote significant differences between the groups (*p* < 0.05).

Figure 1 shows the means and standard errors of the frequency change detection thresholds in musician and non-musician groups under three different stimulus conditions. The mean frequency thresholds were higher (poorer performance) in non-musicians (Stim 1: *M* = 0.72%; Stim 2: *M* = 0.62%; Stim 3: *M* = 0.84%) than in musicians (Stim 1: *M* = 0.42%; Stim 2: *M* = 0.40%; Stim 3: *M* = 0.34%).

A two-way mixed ANOVA was used to determine the effects of the Subject Group (between-subject factor) and the Stimulus Condition (within-subject factor). There was a significant effects of Subject Group, with musicians showing a lower threshold than non-musicians [*F*_(1, 21)_ = 12.64, *p* < 0.05, η*p*^2^ = 0.38]. There was no significant effect of Stimulus Condition [*F*_(2, 42)_ = 0.63, *p* > 0.05, η*p*^2^ = 0.03] nor a significant interaction between Stimulus Condition and Subject Group [*F*_(2, 42)_ = 0.11, *p* > 0.05, η*p*^2^ = 0.01].

### EEG results

**Figure 2:**
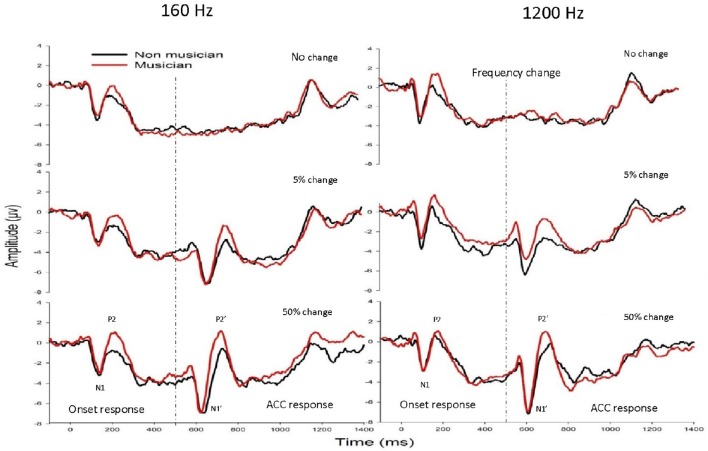
**The grand mean waveforms at electrode Cz from musicians (black traces) and non-musicians (red traces) for 160 Hz (left panel) and 1200 Hz (right panel) with a frequency change of 0% (upper subplots), 5% (middle subplots), and 50% (bottom subplots)**. The onset LAEP and the ACC are marked in one of these plots. There is no ACC when there is no frequency change.

Figure 2 shows the mean waveforms between musicians (black traces) and non-musicians (red traces) for 160 Hz (left panel) and 1200 Hz (right panel) with a frequency change of 0% (top), 5% (middle), and 50% (bottom). Two types of LAEP responses were observed: one with a latency of ~100–250 ms after the stimulus onset and the other occurring 100–250 ms after the acoustic change, respectively, with the former being the onset LAEP or N1-P2 complex and the latter being the ACC or N1′-P2′ complex.

#### Onset LAEP

**Figure 3:**
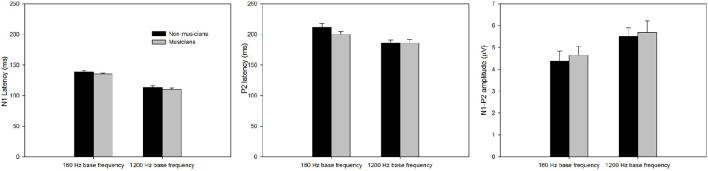
**The onset LAEP measures (N1 latency, P2 latency, and N1-P2 amplitude) for musicians and non-musicians**. The error bars indicate standard errors of the means.

Figure 3 shows the onset LAEP measures (N1 latency, P2 latency, and N1-P2 amplitude) for musicians and non-musicians. The error bars indicate standard errors of the means. As shown in the figure, the onset LAEPs appear to be similar for the tones with three frequency changes (top, middle, and bottom) at each base frequency, because they are evoked by the onset of the same tone regardless of the acoustic change inserted in the middle of the tone. This is an indication of the high repeatability of the LAEP. Compared to non-musicians, musicians have shorter latencies for N1 and P2 for base frequency of 160 Hz and shorter N1 latency for base frequency of 1200 Hz as well as a larger N1-P2 amplitude. The onset LAEP peak latencies tend to be shorter and amplitudes greater for base frequency of 1200 Hz than 160 Hz.

A mixed 2 × 2 repeated ANOVA (Subject Group as the between-subject factor and Base Frequency as the within-subject factor) was performed. The data for different frequency change magnitude (0, 5, and 50%) for each base frequency were averaged since there was no difference in the onset LAEP evoked by these stimulus conditions. Although musicians do show a shorter latency of N1 and P2 and a larger N1-P2 amplitude, the difference did not reach statistical significance (*p* > 0.05). There was a significant main effect of Base Frequency for N1, P2 latency, and N1-P2 amplitude (*p* < 0.05). The 1200 Hz base frequency evoked an onset LAEP with a shorter N1 latency, shorter P2 latency, and larger N1-P2 amplitude.

#### Acoustic change complex

**Figure 4:**
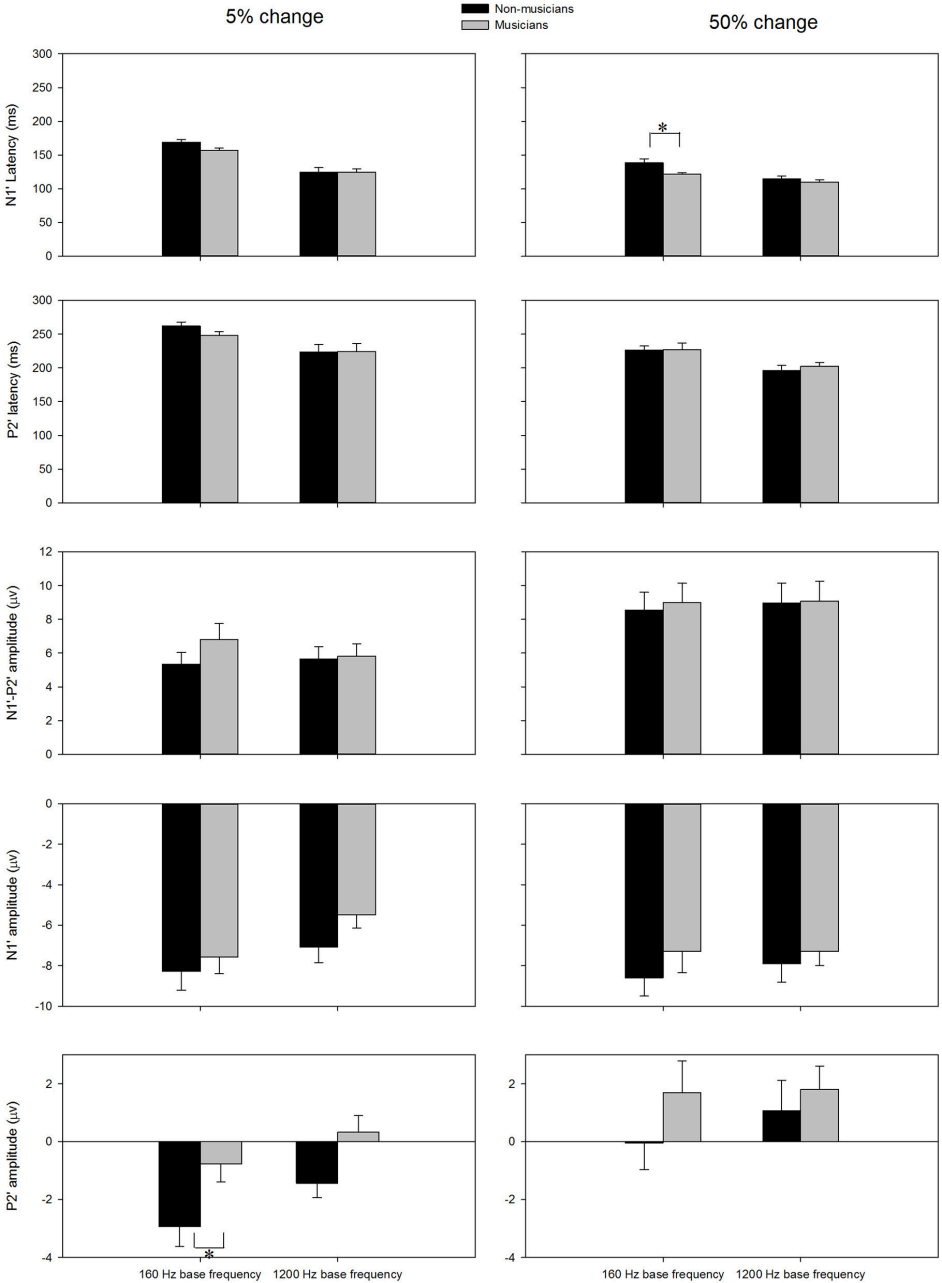
**The ACC measures (N1′ latency, P2′ latency, N1′ amplitude, P2′ amplitude, and N1′-P2′ amplitude) for musicians and non-musicians**. The error bars indicate standard errors of the means. Asterisk denote significant differences between the groups (*p* < 0.05).

The general morphologies of the ACC were similar to those of the onset LAEP, but the amplitude of the ACC appeared to be bigger than the onset LAEP. The ACC occurs only when there is a frequency change in the tone but not when there is no frequency change (Figure 2). Table 1 shows the means and standard deviations of the ACC measures. Musicians have shorter N1′ latency, larger P2′ amplitude, and larger N1′-P2′ amplitude for both base frequencies with both 5% and 50% changes. The ACC amplitudes are bigger and peak latencies shorter for 50% frequency change than for 5% change. The frequency changes at the base frequency 1200 Hz evoked shorter latencies than those at 160 Hz. Figure 4 shows the ACC measures (N1′ amplitude and latency, P2′ amplitude and latency, and N1′-P2′ amplitude) for musicians and non-musicians. The error bars indicate standard errors of the means.

**Table 1:** **ACC measures from musicians and non-musicians**.

**Base frequency (HZ)**	**Change (%)**	**Non-musician**					**Musician**				
		**N1′ latency (ms)**	**N1′ amplitude (μV)**	**P2′ latency (ms)**	**P2′ amplitude (μV)**	**N1′P2′ amplitude (μV)**	**N1′ latency (ms)**	**N1′ amplitude (μV)**	**P2′ latency (ms)**	**P2′ amplitude (μV)**	**N1′P2′ amplitude (μV)**
160	5	169.25 ± 14.50	−8.28 ± 3.21	261.92 ± 20.61	−2.94 ± 2.35	5.34 ± 2.43	156.92 ± 13.17	−7.57 ± 2.88	247.92 ± 19.45	−0.77 ± 2.16	6.80 ± 3.34
	50	138.92 ± 19.39	−8.59 ± 3.17	226.00 ± 21.40	−0.04 ± 3.20	8.55 ± 3.68	122.08 ± 6.71	−7.29 ± 3.67	226.67 ± 35.72	1.70 ± 3.78	8.99 ± 3.95
1200	5	125.00 ± 23.27	−7.08 ± 2.65	223.25 ± 38.82	−1.44 ± 1.71	5.64 ± 2.59	124.67 ± 17.97	−5.48 ± 2.27	224.33 ± 40.23	0.33 ± 1.98	5.81 ± 2.58
	50	114.83 ± 15.33	−7.90 ± 3.11	195.92 ± 27.96	1.07 ± 3.64	8.98 ± 3.97	110.00 ± 10.51	−7.29 ± 2.46	202.42 ± 19.10	1.80 ± 2.79	9.09 ± 4.01

To explore the effects of Base Frequency (within-subject factor), Frequency Change (within-subject factor), and Subject Group (between-subject factor) on the ACC measures, a 2 × 2 × 2 mixed-model repeated ANOVA was conducted separately for N1′ amplitude and latency, P2′ amplitude and latency, and N1′-P2′ peak-to-peak amplitude. Statistical significance was found for N1′ latency and P2′ amplitude. For N1′ latency, there was a main effect of Base Frequency [*F*_(1, 22)_ = 116.01, *p* < 0.01, η*p*^2^ = 0.84], Frequency Change [*F*_(1, 22)_ = 84.88, *p* < 0.01, η*p*^2^ = 0.79] and significant interaction between Base Frequency and Subject Group [*F*_(1, 22)_ = 5.26, *p* < 0.05, η*p*^2^ = 0.19]. No statistical significance was found in Subject Group [*F*_(1, 22)_ = 2.84, *p* > 0.05, η*p*^2^ = 0.12]. For P2′ amplitude, there was a main effect of Base Frequency [*F*_(1, 22)_ = 6.00, *p* < 0.05, η*p*^2^ = 0.21], Frequency Change [*F*_(1, 22)_ = 22.61, *p* < 0.01, η*p*^2^ = 0.51]. No statistical significance was found in Subject Group [*F*_(1, 22)_ = 3.07, *p* > 0.05, η*p*^2^ = 0.12]. Further, 2 × 2 mixed-model repeated ANOVA tests were conducted to examine the effects of Base Frequency and Subject Group on the P2′ amplitude and N1′ latency separately for 5 and 50% change, respectively. For P2′ amplitude, there was a main effect of Base Frequency [*F*_(1, 22)_ = 11.64, *p* < 0.05, η*p*^2^ = 0.35] and Subject Group [*F*_(1, 22)_ = 6.86, *p* < 0.05, η*p*^2^ = 0.24] for 160 Hz 5% change. No statistical significance was found in P2′ amplitude for 160 Hz 50% change (*p* > 0.05). For N1′ latency, there was a main effect of Base Frequency [*F*_(1, 22)_ = 43.00, *p* < 0.05, η*p*^2^ = 0.66] and Subject Group [*F*_(1, 22)_ = 4.82, *p* < 0.05, η*p*^2^ = 0.18] as well as significant interaction between Base Frequency and Subject Group [*F*_(1, 22)_ = 4.73, *p* < 0.05, η*p*^2^ = 0.18] for the 160 Hz, 50% change condition. No statistical significance was found in N1′ latency for the 160 Hz, 5% change condition. In summary, musicians have shorter N1′ latency for 160 Hz with 50% change and larger P2′ amplitude for 160 Hz 5% change; ACCs for 1200 Hz have a shorter N1′ peak latency and larger P2′ amplitude than for 160 Hz. After adjusting the significance level for conducting multiple ANOVAs, the P2′ amplitude was significantly greater in musicians than non-musicians for 160 Hz 5% change.

#### Comparison between the onset LAEP and the ACC

**Figure 5:**
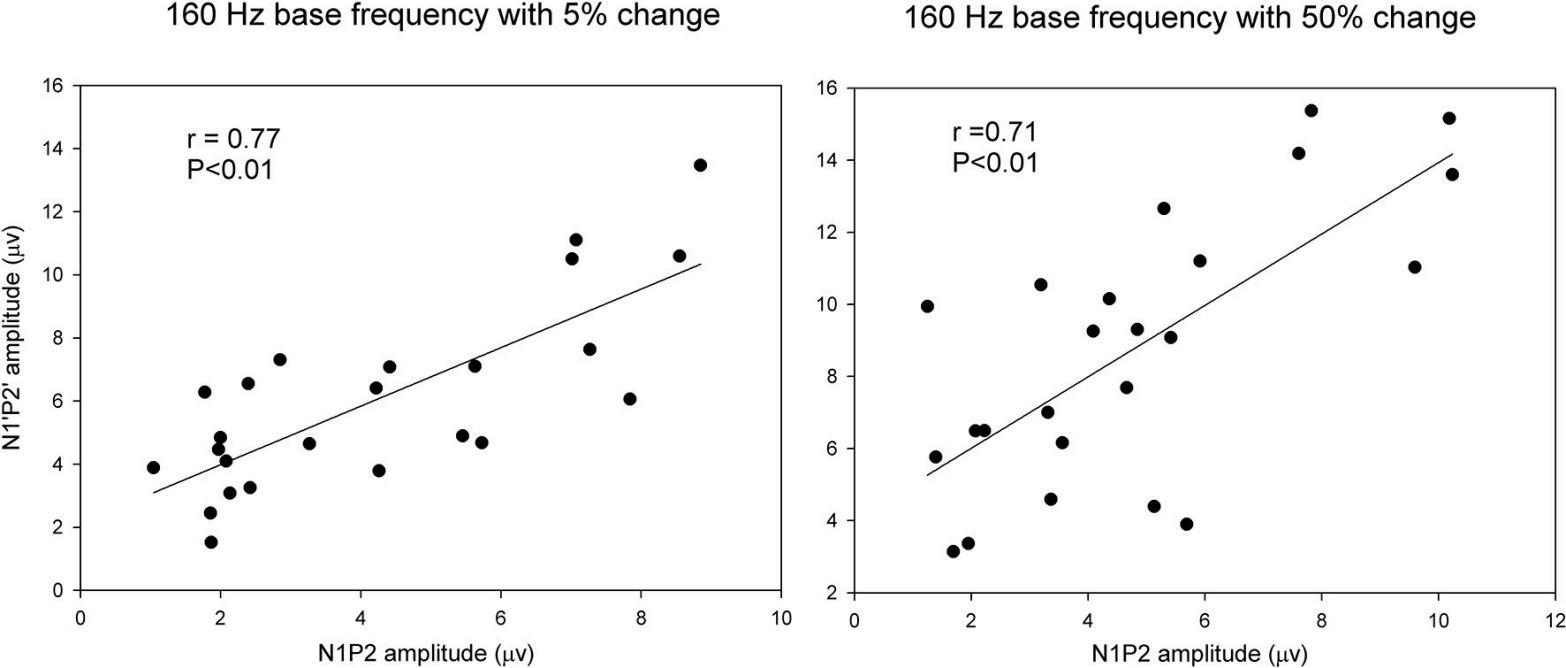
**Scatter plots of ACC amplitude vs. onset LAEP amplitude for the 160 Hz base frequency with 5 and 50% frequency changes**. Data from participants in both musicians and non-musician groups were included. The solid lines show linear regressions fit to the data. The *r*-value for each fit is shown in each panel.

Pearson correlation analyses were performed to determine the correlations between the onset LAEP and the ACC measures. There were significant correlations between the onset LAEP N1-P2 amplitude and the ACC N1′-P2′ amplitude for 160 Hz base frequency with 5% (*r* = 0.77, *p* < 0.01) and 50% change (*r* = 0.71, *p* < 0.01). However, there was no such correlation for 1200 Hz base frequency. Figure 5 shows scatter plots of ACC amplitude vs. onset LAEP amplitude for the 160 Hz base frequency with 5 and 50% frequency changes, respectively. Data from participants in both musician and non-musician groups were included. This finding indicates that participants who display a larger onset LAEP tend to display a larger ACC for the 160 Hz.

#### Comparison between ACC and behavioral measures

The correlation between frequency detection thresholds and the ACC measures were examined. Pearson product moment correlation analysis did not show a significant correlation (*p* > 0.05) between these two types of measures.

## Discussion

The results of this study showed that musicians significantly outperformed non-musicians in detecting frequency changes under quiet and noisy conditions. The ACC occurred when there were perceivable frequency changes in the ongoing tone stimulus. Increasing the magnitude of frequency change resulted in increased ACC amplitudes. Musicians' ACC showed a shorter N1′ latency and larger P2′ amplitude than non-musicians for the base frequency of 160 Hz but not 1200 Hz. The amplitude of the onset LAEP is significantly correlated with that of the ACC for the base frequency of 160 Hz. Below these findings are discussed in a greater detail.

### Evidence of reshaped auditory system in musicians

Numerous behavioral and neurophysiological studies have provided evidence for brain reshaping/enhancement from music training. Behaviorally, musicians generally perform better than non-musicians in various perceptual tasks in music and linguistic domains (Schön et al., 2004; Thompson et al., 2004; Koelsch et al., 2005; Magne et al., 2006; Jentschke and Koelsch, 2009). Anatomically, musicians have enhanced gray matter volume and density in the auditory cortex (Pantev et al., 1998; Gaser and Schlaug, 2003; Shahin et al., 2003; James et al., 2014). Neurophysiologically, musicians display larger event-related potentials (Pantev et al., 2003; Shahin et al., 2003; Koelsch and Siebel, 2005; Musacchia et al., 2008), and their fMRI images show stronger activations in auditory cortex and other brain areas, i.e., the inferior frontolateral cortex, posterior dorsolateral prefrontal cortex, planum temporale, etc., as well as altered hemispheric asymmetry when evoked by music sounds (Pantev et al., 1998; Ohnishi et al., 2001; Schneider et al., 2002; Seung et al., 2005). These differences in brain structures and function are more likely to arise from neuroplastic mechanisms rather than from their pre-existing biological markers of musicality (Shahin et al., 2005). In fact, evidence showed that there is a significant association between the structural changes and practice intensity as well as between the auditory event-related potential and the number of years for music training (Gaser and Schlaug, 2003; George and Coch, 2011). Longitudinal studies that tracked the development of neural markers of musicianship suggested that musician vs. non-musician differences did not exist prior to training; neurobiological differences start to emerge with music training (Shahin et al., 2005; Besson et al., 2007; Kraus and Strait, 2015; Strait et al., 2015).

Musicians perform better than non-musicians in detecting small frequency changes with smaller error rates and a faster reaction time in music, non-linguistic tones, meaningless sentences, native and unfamiliar languages, and even spectrally degraded stimuli such as vocoded stimuli (Tervaniemi et al., 2005; Marques et al., 2007; Wong et al., 2007; Deguchi et al., 2012; Fuller et al., 2014). Micheyl et al. (2006) reported that musicians can detect a 0.15% of frequency difference while the non-musicians can detect 0.5% of frequency difference using 330 Hz as a standard frequency. The authors also reported that the musician advantage of detecting frequency differences is even larger for complex harmonic tones than for pure tone. Kishon-Rabin et al. (2001) reported that the frequency differentiation threshold was ~1.8% for musicians and 3.4% for non-musicians with a standard frequency of 250 Hz (see Figure 1 in Kishon-Rabin et al., 2001).

In the current study, the amount of frequency change at base frequency of 160 Hz can be detected by musicians and non-musicians was 0.42 and 0.72%, respectively, for Stim 1 which is comparable to stimulus condition used in other studies. The difference in the thresholds for frequency change detection between the current study and previous studies may be mainly related to the differences in the specific stimuli or stimulus paradigms used. Most previous studies used trials including standard tone at one frequency and the target tone that has a different frequency. The current study used trials including one standard tone at 160 Hz and one target tone of the same base frequency that contained a frequency change in the middle of the tone. The major difference between these stimulus paradigms is that the behavioral response with the conventional paradigm may reflect that the detection ability of the auditory system for a different frequency plus onset of the different frequency and the response with the current stimulus paradigm may reflect the detection of frequency change only. Additionally, other factors related to stimulus parameters could affect the performance of frequency change detection. Such factors include, but are not limited to, the duration, frequency, whether the stimulus is a pure tone or complex tone, intensity of the stimuli, and the interval between the stimuli in the trials. Despite the differences in the values of thresholds for frequency change detection/frequency discrimination among studies, the current finding that musicians outperform non-musicians in frequency change detection is consistent with that in prior studies (Kishon-Rabin et al., 2001; Micheyl et al., 2006).

This musician benefit extends to the noisy conditions. Although the amount of musician benefits (differences between musicians vs. non-musicians, Figure 1) appears to be greater for Stim 3 compared to other conditions, the difference did not show statistical significance (no significant effect of interaction effect between Stimulus Condition and Subject Group). The lack of group difference in the 3 stimulus conditions suggests that the degree of musicians' benefits in pitch detection is similar in quiet and noisy conditions. The finding that musician benefits exist not only in quiet but also noisy conditions has been reported previously. Fuller et al. (2014) compared musicians and non-musicians in the performance of different types of tasks. The degree of musician effects varied greatly across stimulus conditions. In the conditions involving melodic/pitch pattern identification, there was a significant musician benefit when the melodic/pitch patterns were presented in quiet and noisy conditions. The authors suggested that musicians may be better overall listeners due to better high-level auditory cognitive functioning, not only in noise, but also in general. It would be worthwhile to examine if musician benefits also exist in pitch-based speech and music tasks in noisy conditions in laboratories and real life, which is still controversial with the results from the literature (Parbery-Clark et al., 2009; Strait et al., 2012; Fuller et al., 2014; Ruggles et al., 2014).

### Is musician effect reflected by the ACC?

This is the first study examining the ACC in musician vs. non-musician comparisons. Although the training effects on the ACC have not been reported in previous studies, the training effects of the LAEP have been reported (Shahin et al., 2003; Tremblay et al., 2006). In general, previous studies reported that P2 was enlarged, although there have been some controversies on whether N1 is enhanced in musicians (Shahin et al., 2003, 2005). The previous findings suggest that the P2 is the main component that is susceptible to training. However, the enhanced P2 in musicians does not necessarily reflect the long-term training in musicians only. A short-term auditory training may result in an enlarged P2. Tremblay et al. (2006) examined how a 10-day voice-onset-time (VOT) training changes the LAEP evoked by consonant-vowel speech tokens with various VOTs. The results showed that P2 is the dominant component that is enhanced after this training.

The current study found that the superiority of frequency change detection in musicians can be reflected by ACC measures (P2′ amplitude). N1′ latency was also shorter in musicians than non-musicians, although the difference was not statistically significant. Shorter latencies are thought to reflect faster and more efficient neural transmission and larger amplitudes reflect increased neural synchrony. The shorter N1′ latency and larger P2′ amplitude in musicians may suggest that musicians have a more efficient central processing of pitch changes than non-musicians.

There are some questions that need to be addressed in futures studies. For example, what is the difference between the P2/P2′ enhancements after the long-term vs. short-term training? If the P2/P2′ enhancement in the waveform is the same after long-term and short-term training, would the neural generators for the P2/P2′ be the same? Additionally, it remains a question how much contribution to the enhanced P2/P2′ is from repeated exposures of stimulus trials during the testing (Seppänen et al., 2013; Tremblay et al., 2014)?

### Is musician effect bottom-up or top-down: behavioral and EEG evidence

Sound perception involves “bottom-up” and “top-down” processes that may be disrupted in hearing impaired individuals (Pisoni and Cleary, 2003). “Bottom-up” refers to the early automatic mechanisms (from the peripheral to auditory cortex) that encode the physical properties of sensory inputs (Noesselt et al., 2003). “Top-down” refers to processing (working memory, auditory attention, semantic, syntactical processing, etc.) after passively receiving and automatically detecting sounds (Noesselt et al., 2003).

Previous research provided evidence that the bottom-up process in musicians is enhanced (Jeon and Fricke, 1997; Tervaniemi et al., 2005; Micheyl et al., 2006). For instance, there is a strong correlation between neural responses (e.g., the Frequency Following Response/FFR) from subcortical level and the behavioral measure of frequency perception (Krishnan et al., 2012). Numerous studies have shown enhanced top-down processes in musicians. Behavioral data showed that musicians have better working memory (George and Coch, 2011; Bidelman et al., 2013). The EEG data showed a shorter latency in the P3 response of musicians, which is regarded as the effect of musical experience on cognitive abilities (George and Coch, 2011; Marie et al., 2011). Musicians show a larger gamma-band response (GBR), which has been associated with attentional, expectation, memory retrieval, and integration of top-down, bottom-up, and multisensory processes (Trainor et al., 2009; Ott et al., 2013). Tervaniemi et al. (2005) examined ERPs under passive and active listening conditions. Under passive listening condition, the MMN and P3a, which reflect automatic sound differentiation, did not show difference between musicians and non-musicians. Under active listening conditions during which participants were required to pay attention to the stimuli and identify the deviant stimuli embedded in standard stimuli, the N2b and P3 were larger in musicians than in non-musicians. The authors suggested that musical expertise facilitates effects selectively for cognitive processes under attentional control.

Some researchers have examined the top-down control over bottom-up processes. Using the speech-evoked auditory brainstem response (ABR; Wong et al., 2007; Musacchia et al., 2008; Parbery-Clark et al., 2009; Strait et al., 2010, 2012; Strait and Kraus, 2011; Skoe and Kraus, 2013), Kraus and her colleagues reported enhanced encoding of fundamental frequencies and harmonics in musicians compared with non-musicians; there is a significant correlation between auditory working memory and attention and the ABR properties. Based on these findings, Kraus' group proposed that musicians' perceptual and neural enhancement are driven in a corticofugal or top-down manner. The top-down influence on cortical sensory processing in musicians can also be seen in the stronger efferent fibers linking cortical to subcortical auditory structures and even more peripheral stages of the auditory pathway (Perrot and Collet, 2014). Taken together, a rich amount of evidence suggested that musicians' auditory function is enhanced in a corticofugal top-down driven fashion. In the current study, musicians show a significantly larger P2′ amplitude in the ACC. The N1′ latency in musicians is shorter than non-musicians, although this difference did not reach statistical significance. The shorter N1 and larger P2 was also observed in the onset LAEP of musicians, although the musician vs. non-musician difference did not reach a statistical significance. The N1 has been considered the obligatory response that reflects the sound registration in the auditory cortex and the P2 is not simply an obligatory part of N1-P2 complex; evidence showed that the P2 is a more cognitive component reflecting attention-modulated process required for the performance of auditory discrimination tasks (Crowley and Colrain, 2004). This shortened N1/N1′ and enlarged P2/P2′ in musicians may be the result of a stronger interaction of bottom-up and top-down mechanisms in musicians. Future research with more comprehensive testing (e.g., the use of passive and active listening conditions for EEG and behavioral measures of cognitive function) can be designed to examine or disentangle the role of bottom-up and top-down processing in musician effects on the ACC. The interaction between behavioral outcomes (Hit/Miss) and the features of the ACC will be better revealed in the active listening condition in which the EEG is recorded while the participant performs the behavioral task.

### The ACC vs. the onset LAEP

In the current study, the ACC was evoked by frequency changes contained in pure tones and the onset LAEP was evoked by the onset of the pure tone. The morphologies of onset LAEP and the ACC are very similar. However, several differences between them described below may suggest that the onset LAEP and the ACC involve different neural mechanisms.

First, the onset LAEP is evoked by the stimulus onset. The ACC is evoked by frequency changes not the onset of a new sound, which was removed when the frequency change occurred. Second, the ACC has longer peak latencies, especially for the 5% change. Specifically, ACC measures in Table 1 show that the N1′ and P2′ latencies for the 5% change of base frequency 160 Hz are ~160 and 250 ms, respectively. Onset LAEP measures in Figure 3 show that the N1 and P2 latencies for 160 Hz are ~140 and 210 ms, respectively. The same trend of longer peak latencies for the ACC than for the onset LAEP can be seen for base frequency of 1200 Hz. Third, the ACC has a larger amplitude than the onset LAEP. The amplitude difference of the ACC and the onset LAEP does not look like the result of a higher frequency contained in the tone relative to the base frequency, because the difference of the onset LAEPs evoked by 160 and 1200 Hz is not as dramatic as the amplitude difference between the ACC and onset LAEP. Finally, the amplitude of the ACC is significantly greater when the frequency change is perceptually greater (50 vs. 5%). The ACC amplitude difference between 50 vs. 5% cannot be explained by the difference of the onset LAEP evoked by 160 and 1200 Hz. Hence, the present data suggested that the ACC is evoked by acoustic changes in ongoing stimuli rather than the onset of a new frequency. This supports a previous speculation by some researchers that the ACC is more than a simple onset response (Ostroff et al., 1998).

The distinctions between the ACC and onset LAEP may be caused by different groups of neurons that are responsible for these two different responses, respectively. Animal studies have provided evidence that different groups of neurons in the auditory cortex have functional differences. For instance, in cats' primary auditory cortex, the tonic cells encode information of static auditory signals (e.g., tonal stimulus) with a significant firing increase throughout the stimulus period after a long latency; the phasic-tonic cells encode information of the change of auditory signal during the stimulus period after a medium latency; and the phasic cells (short latency) encode information of rapid change of the auditory signal at onset and offset after a short latency (Chimoto et al., 2002). We speculate that, the onset LAEP is dominantly contributed by the neurons that are sensitive to stimulus onset (e.g., the phasic cells that have shorter response latencies) and the ACC by neurons sensitive to acoustic changes (e.g., the phasic-tonic cells that have longer response latencies). If the onset LAEP and the ACC involves the activation of different groups of cortical neurons, this may suggest that it is important to use stimuli that have acoustic changes with removed onset cues in order to evoke the ACC.

It should be noted that, however, the ACC and onset LAEP may have shared neural mechanisms based on the following findings. Individuals displaying larger onset LAEPs tend to have larger ACCs evoked by frequency changes in 160 Hz base frequency. This finding is consistent with the finding in a previous ACC study in CI users (Brown et al., 2008). Additionally, musician vs. non-musician comparisons in the onset LAEP and the ACC show that musicians have a larger P2/P2′, although the group difference is not significant for the onset LAEP. One possible shared mechanism for these two responses is novelty detection, which may be activated by the stimulus onset that is different from the pre-stimulus quiet period and the frequency change that is different from the previous base frequency. This explanation can be further examined using source mapping in future studies.

It is noted, that the correlation between the onset LAEP and ACC exists only for base frequency 160 not 1200 Hz. This may suggest that the auditory system treats the frequency changes differently for different base frequencies.

### ACC reflects musician benefits

ACC has been suggested by recent studies as a promising tool to show training effects. The current study supports this conclusion with the following findings: (1) there was no ACC when there was no frequency change in the tone, while there was an ACC when there were perceivable frequency changes; (2) the ACC is bigger when the frequency change is perceptually greater (50 vs. 5%); and (3) Musicians had significantly more robust P2′ amplitude compared with non-musicians for 160 Hz base frequency. The lack of correlation between ACC and behavioral measures in the current finding does not exclude the correlation between ACC and behavioral measures. Note, that the ACC in this study was evoked by supra-threshold frequency changes (5 and 50%) rather than the threshold. The use of supra-threshold frequency changes for ACC recordings may be the reason for the failure of observing a significant relationship between the behavioral and ACC measures in the current study. Previous studies have reported that the minimal acoustic change that can evoke the ACC is similar to the threshold for auditory discrimination threshold (He et al., 2012; Kim, 2015). A refined EEG stimulus paradigm (e.g., tones containing a wider range of magnitude of frequency changes), should be used to determine if the perceptual threshold is corresponding to the minimum frequency change that can evoke an ACC in musicians and non-musicians in the future studies.

### ACC vs. MMN

The ACC has the following advantages over another EEG tool that can potentially be used to reflect training effects on perceptual discrimination ability, the mismatch negativity (MMN, Tremblay et al., 1997; Koelsch et al., 1999; Tervaniemi et al., 2005; Itoh et al., 2012). First, the ACC is a more time-efficient measure compared to the MMN (Martin and Boothroyd, 1999). While the MMN requires a large number of trials of stimuli to ensure there are enough trials (e.g., 100–200) of rarely presented stimulus (deviant stimuli) embedded in the frequently presented stimulus (standard stimuli) of the oddball paradigm, ACC is contributed by every trial in the stimulus paradigm. Second, ACC is a more sensitive and efficient evaluation tool than the MMN (Martin and Boothroyd, 1999) due to relatively larger and more stable amplitude. Repeated recording of the ACC has revealed that the ACC is stable and repeatable (Friesen and Tremblay, 2006).

Although the ACC and the MMN are thought to reflect automatic differentiation of sounds at the pre-attentive stage of auditory processing, these responses may involve different neural generators/neurons due to the differences in the stimulus paradigms used to evoke them. Specifically, the ACC is evoked by the acoustic change in the ongoing stimuli. The neurons activated may be the ones that are sensitive to the acoustic change rather than the acoustic onset. In contrast, the MMN reflects the discrepancy between the neural response to the deviant stimuli and the response to the standard stimuli, the neurons activated for the MMN may be the ones that respond to the onset of the deviant stimuli rather than the acoustic change *per-se*. The above speculation can be further confirmed using source mapping in future studies. This speculated difference between the ACC and the MMN may be the reason why there is a significant difference in the ACC between musicians and non-musicians in the current study while there was no difference in the MMN between these two groups in a previous study (Tervaniemi et al., 2005).

## Implications and future work

This study has important implications. First, our findings suggest that long-term music training in individuals with normal auditory systems provides advantages in the frequency tasks that are challenging for hearing impaired patients; the musician benefit is persistent in noisy conditions. However, future studies are still needed to determine if the short-term training in hearing impaired patients, who have different degrees of neural deficits, would result in neurological changes and perceptual improvement in pitch change detection.

Second, the current results also have implications in other populations who have problems in frequency-based perception. For instance, dyslexic children are found to have difficulties discriminating frequency changes that are easily discriminated by normal readers (Besson et al., 2007). Music training would be beneficial to facilitate the brain plasticity toward improving frequency perception and further language perception.

Finally, the ACC can be evoked by frequency changes. The ACC in musicians showed a larger P2′ amplitude than in non-musicians. Because the ACC is recorded without participant's voluntary response, it provides an objective tool to estimate frequency change detection ability and to document training effects.

## Conclusion

To summarize, musicians outperform non-musicians in pitch change detection in quiet and noisy conditions. This musician benefit can be reflected in the ACC measures: the ACC evoked by the frequency change from a base frequency 160 Hz showed a greater P2′ amplitude in musicians than in non-musicians. The ACC displays differences and similarities compared to the onset LAEP, which may suggest these two responses involve different but overlapping neural mechanisms.

## Author contributions

FZ has substantial contributions to the conception and design of the work, the acquisition, analysis, interpretation of data, manuscript preparation, final approval of the version to be submitted, and be accountable for all aspects of the work. CL has substantial contributions to the design of the work, the acquisition, analysis, interpretation of data, drafting the manuscript, final approval of the version to be submitted, and be accountable for all aspects of the work related to data collection and analysis. IT, BE, KW, SC, JX, and QF all have significant contributions to the design of the work, the acquisition, manuscript preparation, final approval of the version to be submitted, and be accountable for their portions of work.

### Conflict of interest statement

The authors declare that the research was conducted in the absence of any commercial or financial relationships that could be construed as a potential conflict of interest.

## Abbreviations

ACC: Acoustic Change Complex
ANOVA: Analysis of Variance
CI: Cochlear Implant
EEG: Electroencephalography
LAEP: Late-latency Auditory Evoked Potential
NH: Normal-Hearing.
